# Comparative transcriptomics of Atlantic *Salmo salar*, chum *Oncorhynchus keta* and pink salmon *O. gorbuscha* during infections with salmon lice *Lepeophtheirus salmonis*

**DOI:** 10.1186/1471-2164-15-200

**Published:** 2014-03-15

**Authors:** Ben JG Sutherland, Kim W Koczka, Motoshige Yasuike, Stuart G Jantzen, Ryosuke Yazawa, Ben F Koop, Simon RM Jones

**Affiliations:** Centre for Biomedical Research, Department of Biology, University of Victoria, Victoria, BC V8W 3N5 Canada; Aquatic Genomics Research Center, National Research Institute of Fisheries Science, Fisheries Research Agency, 2-12-4 Fukuura, Kanazawa, Yokohama, Kanagawa, 236-8648 Japan; Department of Marine Biosciences, Tokyo University of Marine Science and Technology, 4-5-7 Konan, Minato-ku, Tokyo, 108-8477 Japan; Pacific Biological Station, 3190 Hammond Bay Road, Nanaimo, BC V9T 6N7 Canada

**Keywords:** Ecological genomics, Ectoparasite, Host-parasite, Immunity, Inflammation, Iron, Atlantic salmon, Pacific salmon, Sea lice, Transcriptomics

## Abstract

**Background:**

Salmon species vary in susceptibility to infections with the salmon louse (*Lepeophtheirus salmonis*). Comparing mechanisms underlying responses in susceptible and resistant species is important for estimating impacts of infections on wild salmon, selective breeding of farmed salmon, and expanding our knowledge of fish immune responses to ectoparasites. Herein we report three *L. salmonis* experimental infection trials of co-habited Atlantic *Salmo salar*, chum *Oncorhynchus keta* and pink salmon *O. gorbuscha*, profiling hematocrit, blood cortisol concentrations, and transcriptomic responses of the anterior kidney and skin to the infection.

**Results:**

In all trials, infection densities (lice per host weight (g)) were consistently highest on chum salmon, followed by Atlantic salmon, and lowest in pink salmon. At 43 days post-exposure, all lice had developed to motile stages, and infection density was uniformly low among species. Hematocrit was reduced in infected Atlantic and chum salmon, and cortisol was elevated in infected chum salmon. Systemic transcriptomic responses were profiled in all species and large differences in response functions were identified between Atlantic and Pacific (chum and pink) salmon. Pink and chum salmon up-regulated acute phase response genes, including complement and coagulation components, and down-regulated antiviral immune genes. The pink salmon response involved the largest and most diverse iron sequestration and homeostasis mechanisms. Pattern recognition receptors were up-regulated in all species but the active components were often species-specific. *C-type lectin domain family 4 member M* and *acidic mammalian chitinase* were specifically up-regulated in the resistant pink salmon.

**Conclusions:**

Experimental exposures consistently indicated increased susceptibility in chum and Atlantic salmon, and resistance in pink salmon, with differences in infection density occurring within the first three days of infection. Transcriptomic analysis suggested candidate resistance functions including local inflammation with cytokines, specific innate pattern recognition receptors, and iron homeostasis. Suppressed antiviral immunity in both susceptible and resistant species indicates the importance of future work investigating co-infections of viral pathogens and lice.

**Electronic supplementary material:**

The online version of this article (doi:10.1186/1471-2164-15-200) contains supplementary material, which is available to authorized users.

## Background

The global salmon aquaculture industry is challenged by infections with endemic ectoparasitic sea lice such as *Lepeophtheirus salmonis*, *Caligus clemensi*, *C. rogercresseyi* and others. In the Northern Hemisphere, the salmon louse *L. salmonis* has the largest impact [1] and must be properly managed to prevent excessive infections and possible damage to wild salmon populations [2]. Lice disperse as free-swimming nauplii and molt to infective copepodids which attach to a host, develop through later stages and feed on skin and mucus [3]. Motile pre-adult/adult stages are the most damaging to tissues due to large size and aggressive feeding [4]. While lice infections occur regularly on wild salmon [5–7] disease can occur at higher parasite intensities [8] or when hosts are at a sensitive life stage [9, 10]. During infection, the feeding louse elicits a cortisol response in the host [11–13]. Experimental cortisol implants reduce inflammation and increase susceptibility of otherwise resistant coho salmon *Oncorhynchus kisutch*[14] and reduce wound repair of Atlantic salmon *Salmo salar*[15]. Furthermore, louse-derived compounds secreted at the site of attachment can be immunomodulatory (e.g., trypsin-like proteases; prostaglandin E_2_[16–20]) and may facilitate secondary infections.

Salmon lice display increased rates of attraction to and settlement onto susceptible hosts, and are rejected less throughout the infection [21]. The host may incur reduced growth and/or mortality [9]. Susceptibility varies among salmon genera and species, and occurs through host (e.g., behavioral, physiological, immunological) and parasite factors (e.g., physiological, host preference). Coho salmon are considered resistant and rapidly reject lice by innate local inflammation with neutrophils [14]. Also considered resistant are pink salmon *Oncorhynchus gorbuscha* in which early rejection correlates with pro-inflammatory cytokine expression, whereas chum salmon *O. keta* are considered susceptible based on the delay or absence of rejection of *L. salmonis* following laboratory infections [22]. Atlantic salmon are also considered susceptible to infection and responses to the parasite in this host favour a Th2 subset with limited inflammation, leading to chronic infection [23, 24]. Although important for parasite rejection in the resistant host, inflammation and Th1 cellular responses can be costly and lead to self-damage [25, 26]. Balancing resistance with tolerance (e.g., [27]) may play an important role in competent responses to lice.

Heritable variation in susceptibility to *L. salmonis* and *C. elongatus* has been identified in populations of brown trout *S. trutta* and Atlantic salmon [28–30] indicating the potential for selective breeding towards increased resistance in farmed fish [31] and thus reducing requirements for chemical treatments [32]. Identifying genes or pathways involved in competent responses will be important for this process. Variation in the response profiles of candidate cytokines and other immune genes to adult *L. salmonis* was reported in the skin of Atlantic, chum, and pink salmon [33] confirming the importance of skin as an immunological tissue of fish [34]. Here we report a series of controlled exposure trials in which the relative susceptibility of juvenile Atlantic, chum, and pink salmon is confirmed and their physiological responses partially characterised throughout the development cycle of the parasite. In each species, transcriptome profiling of skin and anterior kidney using a recently developed microarray [35] assessed mechanisms elicited over nine days following exposure to the parasite to better understand processes associated with resistance and susceptibility.

## Results

### Infection density and louse development

**Figure 1:**
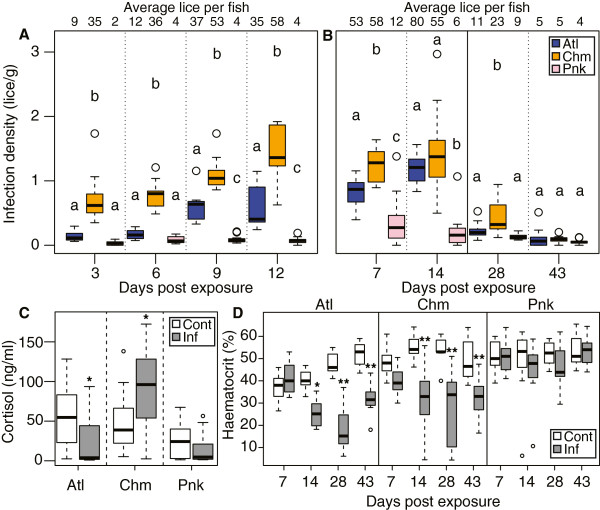
**Infection densities and blood parameters.** Co-habiting Atlantic, chum, and pink salmon were exposed to copepodids in three experimental trials (**(A)** Trial 1, **(B)** Trial 2 and 3), resulting in highest infection density (lice per host weight (g)) in chum, followed by Atlantic, and lowest in pink salmon. Average lice per fish for each condition are presented above the boxplot. Conditions within a day that do not share a letter are significantly different from each other. **(C)** Plasma cortisol (ng/ml) levels in Trial 1 (pooled for days three, six and nine post exposure) indicated elevated cortisol for chum salmon. **(D)** Hematocrit percentages for exposed Atlantic and chum salmon were reduced compared to controls at days 14, 28 and 43, and did not vary for pink salmon. Boxplot displays median and interquartile range, and circles are outliers. *denotes p < 0.05; **denotes p < 0.001.

Infection density (lice per host weight (g)) in Trial 1 was highest in chum salmon, followed by Atlantic salmon and lowest in pink salmon (p < 0.00001) (Figure 1A). Trials 2 and 3 also showed this relative difference, with chum having the highest infection density on day 7 and 28, and chum and Atlantic salmon having equally high infection densities on day 14 (Figure 1B). By day 43 all lice were motile (Additional file 1: Figure S1) and the infection density was reduced and equalized among species. Infection intensity (lice per fish) is also reported in Figure 1 and Additional file 2: Table S1, and follows the same trend as the infection density.

### Fish weights, cortisol, and hematocrit

There was a reduction in weight gain in infected chum salmon relative to controls in Trial 1 (p = 0.012), but no differences were identified in Trials 2 and 3. Average weights for each condition are reported in Additional file 2: Table S1. No significant differences from controls in weight gain were identified for pink or Atlantic salmon in any trials.

No significant temporal effect was noted in the cortisol response, and so data was pooled for all days and compared between infected and control fish for each species (Trial 1; Figure 1C). Plasma cortisol was elevated in infected chum salmon relative to controls (1.75-fold; p = 0.01). Cortisol was not elevated in infected pink or Atlantic salmon relative to control individuals, although a reduction in cortisol occurred in infected Atlantic salmon compared to controls (p < 0.01). This reduction was largely driven by an elevation in control Atlantic salmon cortisol at 9 days post exposure (dpe).

Hematocrit was reduced in Trial 2 and 3 at 14, 28 and 43 dpe in infected Atlantic and chum salmon (p < 0.01; Figure 1D). Infected pink salmon hematocrit did not differ significantly from control individuals.

### Multiple species utility of microarray

Initial normalization of anterior kidney data from all species indicated the largest difference in transcriptome profiles occurred at the genus and species level (principal components analysis PC1 = 64.91% and PC2 = 17.71%; Additional file 3: Figure S2A), which would include species-specific differences in basal gene expression and probe hybridization efficiency. As a result, all species and tissues were separately normalized and comparisons between species were indirect (analysis was performed within a species then results compared across species). Normalized histograms (*not shown*) and the number of probes passing quality control filters for each species were similar (18096, 16716, and 16458 for Atlantic, chum, and pink salmon skin, respectively). Most of the annotated genes expressed in any one species were detected in all three species (Additional file 3: Figure S2B). However, to confirm species differences in expression profiles, qPCR was used to validate hybridization results [36] by using primers with approximately equal efficiency for all three species (Additional file 4: Table S2).

### Anterior kidney transcriptomics: systemic responses of Atlantic, chum, and pink salmon

**Figure 2:**
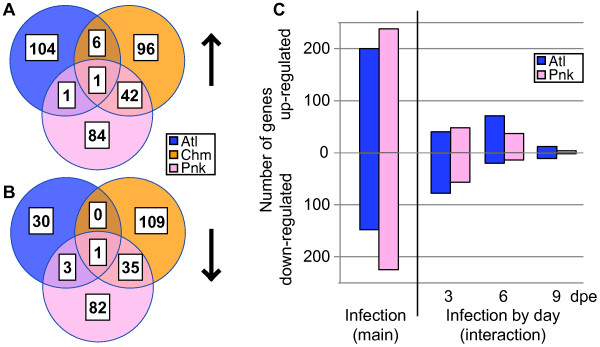
**Anterior kidney transcriptome responses.** At six days post exposure, anterior kidney responses varied depending on host species in either the **(A)** up-regulated or **(B)** down-regulated gene lists. Consistently more genes were shared between chum and pink salmon than with Atlantic salmon, including up-regulation of *hepcidin-1*, *prostaglandin E synthase 3* and down-regulation of antiviral response genes. **(C)** Most genes were identified with a main effect of infection (response independent of day post exposure). Genes responding with a time by infection interaction (response dependent on day) were mainly identified early in the response, at day three or six.

The louse infection affected gene expression in the anterior kidney of all species (Figure 2). Atlantic and pink salmon responses were profiled over nine days at three time points (3, 6, 9 dpe), but chum salmon were only profiled at 6 dpe. For each species, infection class (control or infected), and day combination, 9–11 individuals were profiled (i.e. total Atlantic, chum, and pink salmon anterior kidney samples profiled = 57, 20, 60, respectively). To keep sample numbers similar among species, the initial analysis was restricted to 6 dpe for all species. A similar number of uniquely annotated genes were differentially expressed at 6 dpe, and these were largely species-specific although some similarities were identified between pink and chum salmon (Figure 2A-B).

Time course data for Atlantic and pink salmon anterior kidney indicated the majority of differentially expressed genes responded similarly across the first nine days of infection (main effect infection), while a smaller subset responded differently depending on day (time by infection interaction; Figure 2C). Uniquely annotated genes responding in a similar manner across all days included 200 up- and 148 down-regulated genes in Atlantic salmon, and 238 up- and 225 down-regulated genes in pink salmon. For both species, most time-dependent genes were specific to the early days of the infection (Figure 2C) and these genes were almost entirely exclusive to each species (Additional file 5: Table S3).

The protein folding response was up-regulated in the anterior kidney of all species (p < 0.01; Table 1). Unfolded proteins are typically an indicator of cellular stress (see [37]). Other up-regulated indicators of cellular stress included *stress-induced phosphoprotein 1* (Atlantic and pink), *damage inducible transcript 4-like* and *stress-associated ER protein 1* (chum), *growth arrest and DNA-damage induced protein gadd45 beta* (pink), *programmed cell death protein 10* (pink), *apoptosis induced factor 2* (pink), *stress-70 protein* (Atlantic) (Additional file 6: Figure S3). Cyclin-dependent kinase inhibitors promote cell cycle arrest at G1 phase [38]. In Atlantic salmon, *cyclin-dependent kinase 4 inhibitor b* (*cdkn2b*) was highly up-regulated at day 6 and 9, and *cyclin-dependent kinase inhibitor 1c* was also up-regulated. *cdkn2b* induces cell cycle arrest in response to TGF-β [39]. These genes were not differentially expressed in chum salmon, although the Pacific salmon specifically up-regulated *cyclin-dependent kinase inhibitor 1*, albeit not to the same extent as *cdkn2b* in Atlantic salmon (Additional file 6: Figure S3). Energetic costs of the infection, whether from rejection or tolerance mechanisms are reflected in the enrichment of energy usage (ATP-binding p < 0.05; Table 1) in Atlantic and chum salmon up-regulated lists.

**Table 1:** **Gene ontology enrichment of systemic responses to lice infection**

		GO term	No. genes	p-value	Fold enrich.
***Atl up***	BP	Protein folding	13	1.39E-05	4.7
	MF	ATP binding	36	3.37E-04	1.8
	MF	Metallopeptidase activity	7	1.00E-02	3.8
***Atl down***	BP	Amine metabolic process	13	6.58E-05	4.0
	MF	Enzyme inhibitor activity	7	2.70E-03	4.9
***Chm up***	BP	Amine metabolic process	14	2.20E-05	4.2
	BP	Protein folding	8	4.10E-03	3.9
	MF	ATP binding	22	2.62E-02	1.6
***Chm down***	BP	Immune response	11	3.36E-04	4.0
	BP	Response to virus	5	3.50E-03	7.7
	BP	Antigen processing and presentation of peptide or polysaccharide antigen via MHC class II	4	3.08E-04	27.1
	MF	Carbohydrate binding	8	4.10E-03	3.9
***Pnk up***	BP	Protein folding	13	7.90E-05	4.0
***Pnk down***	BP	Nitrogen compound biosynthetic process	15	8.42E-05	3.5
	BP	Heme biosynthetic process	4	1.30E-03	16.9
	BP	Erythrocyte development	4	1.30E-03	16.9
	BP	Response to virus	5	2.05E-02	4.7
	BP	Immune system process	16	3.37E-02	1.8

Selected Gene Ontology categories enriched in responses occurring generally over the nine days of infection in Atlantic (Atl), chum (Chm), and pink (Pnk) salmon anterior kidney. BP, biological process; MF, molecular function.

**Figure 3:**
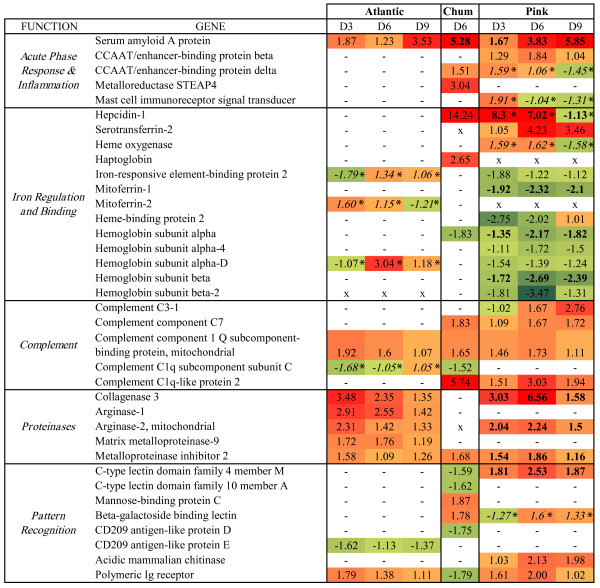
**Comparative gene expression responses in key functional groups.** Differentially expressed genes in the anterior kidney involved in the acute phase response, iron regulation, complement activity, proteinase activity, or pattern recognition are displayed with linear fold change values for each day (D3-D9) and colored by fold change relative to controls (green = down-regulated; red = up-regulated). Bold values indicate highly significant main effect of infection (p < 0.0001), asterisks indicate significant time by infection interaction, and italics indicates no significant main effect (significant interaction only). A hyphen indicates no significant difference identified and an ‘x’ indicates no probe passing quality control for the species.

While expression of the acute phase protein *serum amyloid A* was increased in all species, pink salmon in particular and to a lesser extent chum salmon up-regulated other components of the acute phase response, including *CCAAT/enhancer binding proteins*, and complement genes including *complement component c7* (pink and chum) and *complement component c3* (pink only; Figure 3). All three species also showed differential expression of components of the coagulation cascade (Additional file 6: Figure S3), although the genes involved differed among the species.

**Figure 4:**
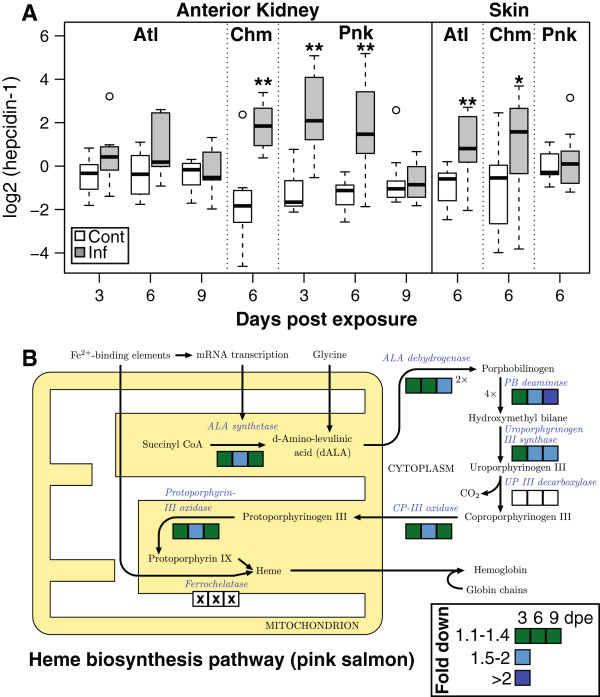
**Species and tissue expression of iron regulation mechanisms. (A)** *hepcidin-1* was highly up-regulated in the anterior kidney of pink salmon early in the infection period (day three and six only). Chum salmon highly increased *hepcidin-1* expression in the anterior kidney and more moderately in the skin. Atlantic salmon up-regulated *hepcidin-1* in the skin but not in the anterior kidney (*hepcidin-1* data shown is from qPCR). Boxplot displays median and interquartile range, and circles are outliers. *denotes p < 0.05; **denotes p < 0.001. *Hepcidin-1* induction was a general response to the infection, whereas other iron homeostasis mechanisms, such as **(B)** reduction of expression of the heme biosynthesis pathway, were specific to pink salmon. Boxes indicate fold change for day 3, 6 and 9 post infection; an x indicates no probe for analysis, and an empty box indicates no significant difference in expression (heme biosynthesis transcripts shown are from microarray data). Image adapted from: *Wikimedia Commons “Heme synthesis” Creative Commons Attribution-ShareAlike 3.0 Unported.*

Iron regulation was induced alongside up-regulation of complement/acute phase response in pink salmon. The main regulator of iron homeostasis, *hepcidin-1* was highly up-regulated in both chum and pink salmon (Figures 3 and 4A). In pink salmon this induction was specific to 3 and 6 dpe, with expression returning to baseline by 9 dpe. Genes involved in scavenging iron from blood and sequestering in tissues including *serotransferrin-2* and *haptoglobin* were up-regulated in pink and chum salmon, respectively. Pink salmon suppressed heme biosynthesis through suppression of six of the seven enzymes in the pathway (Table 1; Figure 4B). Pink salmon up-regulated the heme-recycling *heme oxygenase* specifically at 3 and 6 dpe, and down-regulated several hemoglobin subunits (n = 5), as well as *mitoferrin-1* and *heme binding protein 2* (Figure 3). Both chum and pink salmon induced iron regulatory mechanisms, although some components were specific to pink salmon (e.g., suppression of heme biosynthesis).

Innate pattern recognition receptors may be involved in recognizing the parasite or cell damages, and subsequently inducing appropriate response mechanisms. Pattern recognition receptors were induced in all species but the active components were species-specific (Figure 3). Up-regulation of *c-type lectin domain family 4 member M* occurred only in pink salmon (p < 0.0001), whereas up-regulation of *mannose-binding protein C* occurred only in chum salmon. *Beta-galactoside binding lectin* up-regulation occurred in pink and chum salmon at 6 dpe. As identified previously [24], *polymeric Ig receptor* increased in expression for Atlantic salmon. Here, pink salmon also up-regulated this transcript, whereas expression was down-regulated in chum salmon (Figure 3). However, an additional *polymeric Ig receptor* probe indicated down-regulation in pink salmon (*data not shown*). Specific to pink salmon was the induction of *acidic mammalian chitinase*, previously identified as one of the highest up-regulated genes in juvenile pink salmon responding to salmon lice [10]. The protein encoded by this gene has chitinase activity [40], and plays a role in allergic inflammation [41].

Suppression of antiviral response gene expression was characteristic of the anterior kidney of both pink and chum salmon. Pink salmon down-regulated seven interferon-induced genes such as *interferon-induced GTP-binding protein Mx*, *interferon regulatory factor 1, 3, and 7*, three tripartite motif-containing genes and *signal transducer and activator of transcription 1* (Additional file 7: Figure S4). Many of these genes were also suppressed in chum salmon. Enrichment was found in the down-regulated lists of both chum and pink salmon for response to virus (p < 0.05; Table 1). Atlantic and chum salmon both down-regulated several chains of the MHC class II antigen presentation machinery (Additional file 7: Figure S4).

Considering the important immunomodulatory role of prostaglandin E_2_ in the louse-salmon interaction [17], it is interesting to note that *prostaglandin E synthase 3* was up-regulated in all species (Figure 3). However, the role of this transcript is unclear because in addition to generating prostaglandin E_2_, this enzyme is a co-chaperone of HSP90 and the unfolded protein response is activated in all species (Table 1). In addition, a prostaglandin inactivator, *15-hydroxyprostaglandin dehydrogenase* [*NAD+*] was suppressed at 3 and 6 dpe in pink salmon, and at 6 dpe in chum salmon (Additional file 6: Figure S3).

Differential expression of several components of cell-mediated immunity was evident in Atlantic salmon responses, including the up-regulation of the highly inflammatory *leukotriene B4 receptor* and *high affinity interleukin-8 receptor B*, both specific to Atlantic salmon (Additional file 6: Figure S3 and Additional file 7: Figure S4). Chum salmon increased expression of the *Ig mu chain region membrane bound form*, and *CD276 antigen* (Additional file 7: Figure S4).

Metalloproteinase expression is typically induced in response to salmon lice [10, 23, 24]. Atlantic salmon in the present study up-regulated several metalloproteinases: *collagenase-3* (*mmp13*), *matrix metalloproteinase-9*, and *arginase-1* and −*2* (Figure 3). Only *mmp13* and *arginase-2* were up-regulated in pink salmon, and *mmp13* was one of the highest up-regulated genes for pink salmon anterior kidney (Figure 3). Interestingly, chum salmon did not increase expression of any of these metalloproteinases, although *metalloproteinase inhibitor 2* was up-regulated in all species.

### Local transcriptomic responses of Atlantic, chum, and pink salmon

In the microarray analysis of the skin (by sampling pectoral fin), all species were profiled at six days post exposure, with 9 or 10 individuals used for each species and infection class combination (i.e. total Atlantic, chum, and pink skin samples = 18, 20, 19, respectively). Differential expression was mainly identified in chum salmon, with 44 up-regulated genes, and 86 down-regulated genes. There were only four probes differentially expressed in pink salmon skin (two probes without annotation, *suppressor of fused homolog* and *guanidinoacetate N-methyltransferase*) and no differential expression was found in Atlantic salmon skin.

Genes up-regulated in chum skin were involved in cell death (6 genes; p = 0.012) and those down-regulated were involved in immune response (9 genes; p < 0.001). The *complement component C7* gene was up-regulated (Additional file 8: Figure S5) as in the anterior kidney. Expression of *interleukin-20 receptor alpha chain* was down-regulated. IL-20 signalling through signal transducer and activator of transcription-3 generates potent cutaneous inflammation [42]. Cell proliferative genes were also up-regulated, such as *fibroblast growth factor-binding protein 1*, a keratinocyte mitogen up-regulated after skin injury in epithelial cells [43] and *adseverin*, a regulator of chondrocyte proliferation and differentiation [44] (Additional file 8: Figure S5). However, also up-regulated was *growth arrest and dna-damage-inducible protein gadd45 beta*, which is induced by genotoxic agents or apoptotic cytokines and has a role in reducing proliferation [45]. Furthermore, induction of *thioredoxin* was identified, which is involved in protection from reactive oxygen species-induced stress. Interestingly, the highest up-regulated annotated gene was *FK506-binding protein 5*, which was also up-regulated in the anterior kidney of all species (Additional file 6: Figure S3). Similar to the anterior kidney of pink and chum salmon, many antiviral components were suppressed in chum salmon skin (Additional file 8: Figure S5). The local and systemic responses of chum salmon indicated some consistencies between tissues, and consistencies were more frequently observed for down-regulated genes (38 of 86 in the anterior kidney) than for up-regulated genes (7 of 43 in the anterior kidney).

### Microarray validation and cytokine exploration by quantitative PCR

All genes tested with quantitative PCR (qPCR) had the same direction of fold change as was found differentially expressed in microarray analysis. Correlation of qPCR and microarray data indicated reliability of estimates for each species: the average R squared ± standard deviation for anterior kidney genes was 0.648 ± 0.224 (n = 9 gene/species comparisons; Additional file 9: Figure S6). The trends identified for *hepcidin-1*, *collagenase-3*, and *15-hydroxyprostaglandin dehydrogenase* [*NAD+*] in the anterior kidney of all three species were confirmed with qPCR, including the unchanging expression of *collagenase-3* in chum salmon (Figure 4A and Additional file 10: Figure S7).

**Figure 5:**
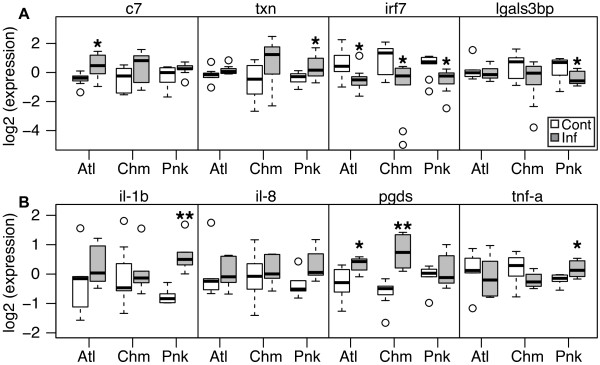
**Local expression of immune genes in fin by qPCR.** Local tissue expression was profiled in all three species at six days post exposure. Genes displayed in **(A)** were selected based on microarray analysis, and in **(B)** were selected based on previous analyses. As per the microarray analysis, all genes were normalized within a species and therefore the only valid comparison to make is between control (white) and infected (grey) within a species. In all three species, *interferon response factor 7* (irf7) was down-regulated. Pink salmon up-regulated *thioredoxin* (txn) and down-regulated *galectin 3-binding protein* (lgals3bp), and Atlantic salmon up-regulated *complement C7* (c7). Both Atlantic and chum salmon up-regulated *prostaglandin D synthase* (pgds), and pink salmon was the only species to induce pro-inflammatory cytokine *interleukin-1 beta* (il-1b), and slightly *tumor necrosis factor alpha* (tnf-a), although the fold change was low (1.3-fold). Boxplot displays median and interquartile range, and circles are outliers. *denotes p < 0.05; **denotes p < 0.001.

Occasionally, differential expression of certain genes was indicated by qPCR but not by the microarray analysis, presumably because of the multiple test correction applied to the microarray. Measured by qPCR, up-regulation of *hepcidin-1* occurred in Atlantic and chum salmon skin (Figure 4A). Also, *complement C7* up-regulation occurred in Atlantic salmon skin (Figure 5A) but not in pink salmon, despite up-regulation in pink salmon anterior kidney. Additionally, *interferon response factor 7* was identified as down-regulated by qPCR in skin of all species including Atlantic salmon (Figure 5A). qPCR identified down-regulation of *galectin-3-binding protein* and up-regulation of thioredoxin in the skin of pink salmon, whereas these genes did not pass significance testing in chum salmon (p = 0.06). When tested with qPCR, *15-hydroxyprostaglandin dehydrogenase* [*NAD+*] was found to be suppressed early in all species (3 or 6 dpe; Additional file 10: Figure S7B), not just in pink and chum salmon as identified with the microarray. Use of qPCR to validate the microarray confirmed that the trends identified in the array analysis were largely correct and not confounded by species differences in probe hybridization efficiencies.

Exploratory qPCR of targets not on the microarray identified up-regulation in pink salmon skin of pro-inflammatory cytokine *interleukin-1 beta* (2.6 fold; p = 0.001), as well as a slight elevation in *tumor necrosis factor alpha* (1.3 fold; p < 0.05; Figure 5B). These genes were not differentially expressed in the other species. *Interleukin-8* was not differentially expressed in skin of any species. Increased expression of *prostaglandin D synthase* occurred in the skin of Atlantic (FC = 1.6) and chum salmon (FC = 2.6), but not pink salmon. None of these genes were up-regulated in the anterior kidney of any species, although *tumor necrosis factor alpha* was down-regulated in chum salmon anterior kidney (FC = 1.8; p < 0.007), and *prostaglandin D synthase* was down-regulated in Atlantic salmon anterior kidney (FC = 1.7; p < 0.002).

## Discussion

The present work shows that when co-habited and subjected to identical copepodid exposures, chum salmon become infected with higher densities (lice per host weight (g)) of salmon lice *Lepeophtheirus salmonis* than do Atlantic or pink salmon. The higher infection density on chum compared to pink salmon was previously identified [22] and the inclusion of Atlantic salmon here provides more information on the susceptibility spectrum of Pacific and Atlantic salmon. We conclude that juvenile pink salmon are resistant whereas juvenile Atlantic and particularly chum salmon are susceptible. This comparative infection system permitted the analyses of hematological parameters in addition to local and systemic transcriptomic responses to identify mechanisms associated with this susceptibility variation.

Differences in infection density among species were observed three days post exposure indicating either a) the rapid onset of an innate effector mechanism in pink salmon or b) greater affinity of infective copepodids for chum and Atlantic salmon through behavioral or chemical cues (e.g., [5, 46, 47]) or c) a combination of these processes. While further research is required to better understand the relative affinity of *L. salmonis* for Pacific salmon species, it is understood that pathology occurs throughout the infection, with most damage occurring after the lice molt to adult stages [4]. Here, the consequences of elevated infection densities on chum and Atlantic salmon were reflected in elevated plasma cortisol (chum), reduced weight gain (chum), and reduced hematocrit (chum and Atlantic). Hematocrit reduction in exposed chum and Atlantic salmon and no significant effect in pink salmon confirms previous observations in these species, and this reduction was also noted in sea trout *S. trutta*, and in sockeye salmon *O. nerka* infected by lice [4, 22, 48, 49]. In these studies, the reduced hematocrit was related to infection intensity and possibly indicative of a microcytic anemia induced by lesions in the skin caused by feeding parasites, leading to fluid loss. Elevated plasma chloride levels, frequently reported during *L. salmonis* infections, are associated with altered osmoregulatory capacity caused by feeding behavior of the larger and more aggressive motile stages [4, 48, 50]. Plasma cortisol was elevated in chum salmon infected with chalimus stages, confirming an earlier report for chum salmon infected with motile *L. salmonis* stages [22]. Similarly, other studies have identified elevated plasma cortisol in Atlantic salmon coincident with the first appearance of motile *L. salmonis* stages [12, 46]. It is possible that the earlier induction of cortisol in the present study as well as elevated cortisol in control Atlantic salmon could be due to stresses of co-habitation with mixed species. The apparent increase in infection density in all three species at day 9 and 12 in Trial 1 was due to the shedding into the anaesthetic bucket of copepodids that were incompletely attached via frontal filament on days 3 and 6, as observed previously [14]. This comparative laboratory infection model has provided a reliable tool with which to explore the transcriptomic basis of host responses to *L. salmonis* among salmon species displaying resistant and susceptible phenotypes.

Cytokine profiling and functional analysis of gene lists indicated that inflammation and the acute phase response (APR) were important response mechanisms following exposure to *L. salmonis*. The pro-inflammatory cytokines *interleukin-1 beta* and *tumor necrosis factor alpha* were induced only in the skin of pink salmon. *Interleukin-1 beta* promotes the T helper 17 (Th17) cell response, further indicating the importance of this function in responses to salmon lice (e.g., [23]). Th17 responses induce inflammation during host defense against bacterial or fungal infection, but can also play a role in tissue pathology and autoimmunity [51]. In the present work, the APR was recognized in all species by the increased expression of *serum amyloid A* during infections [52]. Other identified acute phase proteins were induced in pink and chum salmon, including common and species-specific responses. In pink salmon, with the exception of *serum amyloid A*, the APR decreased by day 9, whereas in Atlantic salmon, the onset of *serum amyloid A* expression appeared delayed. Atlantic salmon previously have been shown to respond to lice after one to three dpe with induction of genes involved in the acute phase response [24, 33]. Up-regulation of complement components was also identified as a general response in all three species. Complement plays a role in chemotaxis, opsonization and vascular permeability, and can be induced alongside acute phase responses [52]. The up-regulation of *c3* solely in pink salmon indicated increased capacity for innate immunity through complement activation via classical, alternative and lectin pathways [21]. Coagulation is an important first step of tissue repair following injury [53] and the identification of these functions mainly in pink and chum salmon suggested they are part of a general response to the infection. Infections with *L. salmonis* are known to elicit inflammation at attachment sites on the skin and that these reactions differ considerably among host species. Reactions to *L. salmonis* are minimal in the skin of Atlantic salmon and pronounced in coho salmon [14, 46]. It has therefore been postulated that the capacity to mount an inflammatory response at the site of parasite attachment is an indicator of resistance and more explicitly, that inflammation is an important defence mechanism in promoting early rejection of parasites [8, 22]. Our data confirm the occurrence of general and species-specific indicators of cutaneous and systemic inflammation following exposure to *L. salmonis*. Furthermore in pink salmon, the cutaneous production of proinflammatory cytokines, systemic APR and enhanced capacity for complement function may help explain the low levels of infections compared with those on chum and Atlantic salmon.

Early infection with *L. salmonis* was associated with changes in the expression of genes associated with iron regulation and binding. The affected pathways tended to be species-specific; haptoglobin was only up-regulated in chum salmon and the majority of dysregulated genes was observed in pink salmon. The fold change of *hepcidin-1* up-regulation in pink and chum salmon was the highest of all genes measured in this study. *Hepcidin-1* regulates iron homeostasis by preventing export of iron from cells into the blood [54] and is induced by *interleukin-6* during inflammation [55], by endoplasmic reticulum stress [56] or as part of a type II acute phase response [57]. Both the antimicrobial and iron regulatory roles of *hepcidin-1* are evolutionarily conserved in a broad range of fish species (for review see [58, 59]). For example *hepcidin-1* was induced in the anterior kidney of barramundi *Lates calcarifer* after intraperitoneal injection with lipopolysaccharide [60], in the anterior kidney of miiuy croaker *Miichthys miiuy* after injection with *Vibrio anguillarum*[61] and in the liver of sea bass *Dicentrarchus labrax* from both iron overload and bacterial infection [62]. Here, *hepcidin-1* expression was induced in the anterior kidney of both pink and chum salmon by the louse infection. By day 9 however, *hepcidin-1* expression was back to baseline in pink salmon kidney, coincident with the highest *serum amyloid A* up-regulation. *Hepcidin-1* was also induced in the skin of Atlantic and chum salmon, the most heavily infected species. However, other iron homeostatic components were specific to pink salmon, including up-regulation of heme recycling *heme oxygenase* and iron scavenging *serotransferrin-2*, and down-regulation of hemoglobin subunits and the heme biosynthesis pathway. This suggests that nutritional immunity [56, 63, 64], the sequestration of host nutrients from pathogens may have a role in defence against salmon lice. A highly anemic state is likely not the end result of this protective mechanism, as in Trials 2 and 3 only Atlantic and chum salmon showed significant hematocrit reduction, likely due to breaches in the circulatory system as discussed above. Alternatively, anemia of inflammation is often mild and accompanies changes in iron handling and erythrocyte production and lifespan [65]. Previous work identified an increase in the quantity of cleaved transferrin fragments in the mucus of *L. salmonis-*infected Atlantic salmon, and the authors discussed the possibility of this being due to louse-mediated modulation of the iron sequestration role of transferrin [66]. Both the necessity of iron in the salmon louse diet and the role for sequestration of iron during the host-parasite interaction merit further study.

Tolerance of infection can also be an adaptive alternative to inflammation-based rejection mechanisms by reducing damage to self [67]. Up-regulation of the protein folding response, evident in the anterior kidney of all species during *L. salmonis*, was an indication of cellular protection. Previous work also identified up-regulation of protein folding transcripts in the skin of Atlantic salmon infected with lice at 22–33 dpe [23]. These cellular protective mechanisms in the anterior kidney suggest infection is associated with self-damage induced by reactive oxygen species or other defense mechanisms. Similarly, evidence of increased expression of the antioxidant *thioredoxin* in the skin of pink salmon provided additional support of pro-tolerance mechanisms as overexpression of *thioredoxin* can protect from oxidative stress induced during infection or inflammation in mammals [68]. Enrichment of ATP binding in chum and Atlantic salmon indicates costs are associated with either mechanisms of tolerating infection or responding to infection. We suggest that salmon adopt a species-specific but balanced response to *L. salmonis*, including both resistance and tolerance mechanisms, in which energetic costs are minimized while reducing negative consequences of infection.

Metalloproteinases are important for initiation and resolution of inflammation in teleosts by degrading damaged extracellular matrix prior to tissue remodeling [69] and expression of these genes in response to salmon lice has been recognised in both Atlantic and pink salmon [10, 23, 24]. In the present study, *collagenase-3* and *arginase-2* were up-regulated in the anterior kidney of both pink and Atlantic salmon throughout the infection. The induction of *arginase-1* was specific to Atlantic salmon. This transcript suppresses Th2 cytokine-driven inflammation, an important mediator of ectoparasite defense [67]. Previously, reduced cell proliferation combined with increased metalloproteinase activity was identified in chronic infections of susceptible Atlantic salmon [23] and in *L. salmonis*-sensitive juvenile pink salmon [10]. Here, *cyclin-dependent kinase 4 inhibitor b* was highly up-regulated in Atlantic salmon coincident with multiple metalloproteinases, providing further evidence for this combination in susceptible species. Interestingly, metalloproteinase transcripts were not up-regulated in chum salmon, but the effect of this apparent deficiency with respect to louse susceptibility is not known.

Innate pattern recognition molecules such as lectins can relay information about self damage or danger, and can induce appropriate pathways of defense. Unique to pink salmon was the induction of *c-type lectin domain family 4 member M* (*clec4m*) and *acidic mammalian chitinase* (*amcase*). *clec4m* is a transmembrane pattern recognition receptor involved in cell adhesion and capable of recognizing various divergent pathogens, however, its role in the response to *L. salmonis* is not known. In earlier work, a C-type lectin was more abundant in the mucus of lice-infected Atlantic salmon [66]. It will be interesting to continue to characterize the different lectins induced in different salmon species and their relative conferred protection. The Th2 response mediator and chitin degrading enzyme *amcase* was also one of the most highly up-regulated genes in 0.7 g juvenile pink salmon during salmon lice infection [10]. We suggest these two pattern recognition molecules play a role in the innate defence of juvenile pink salmon to *L. salmonis*, and that additional research is required to determine more precisely their function.

A striking result in both the susceptible chum salmon and the resistant pink salmon was the suppression of many antiviral response genes, including *interferon response factor 3* and *7* and *signal transducer and activator of transcription 1*. Previous work reported suppression of antiviral response genes in Atlantic salmon skin in response to salmon lice (1–10 dpe; [24]). We propose that the suppression is due to an inverse relationship to another component of the immune system. The antiviral response may exert a negative effect on the more suitable immune response, could be energetically expensive or may induce further self damage. An inverse correlation between antiviral (type I interferons, IFN-α and IFN-β) and anti-bacterial/anti-parasitic (type II interferons, IFN-γ) has been identified in human anti-mycobacterial responses [70]. Energetic costs of tissue remodeling during louse infection have been identified in sensitive juvenile pink salmon [10]. Protection from cellular damage was identified in the protein folding response in the anterior kidney of all species responding to the louse infection. Interestingly, the suppression of antiviral immunity transcripts implies a basal surveillance mechanism exists in healthy fish, and this has been referred to as intrinsic antiviral immunity in mammals [71]. The inverse relationship between these components of the immune system also raises important questions concerning the influence of *L. salmonis* infection on host susceptibility to viruses and other intracellular pathogens. An alternate hypothesis to the inverse regulation hypothesis is that the suppression is due to parasite-derived immunomodulatory compounds. The presence of the suppression in the resistant pink salmon at the same time as up-regulation of more suitable immune genes suggests this is not the case. Another possibility is that the down-regulation is due to the cells carrying these antiviral transcripts are mobile and move to another tissue, however, the suppression was identified in both the anterior kidney and the skin, and so this is not likely either. Therefore, we propose that antiviral suppression during a louse infection is a general response to the infection, and is an intrinsic response that occurs from inverse regulation to another component of the immune system.

Few probes were found differentially expressed in skin of Atlantic or pink salmon, despite using the same multiple test correction methods applied to the anterior kidney transcripts. This is probably due to the relatively low infection densities on pink and Atlantic salmon and the use of pectoral fin as a surrogate for skin, regardless of the presence of lice. Previous work found differences in host gene expression between the site of attachment and a distant site on the skin of the same fish [33]. Also, the fin sample included multiple tissue types, thus contributing to variation in the data, and reducing the possibility of finding differentially expressed genes with stringent statistical testing. In contrast to Atlantic and pink salmon, the heavier infection of the chum salmon increased the probability of infection on the fin in all samples with a corresponding increase in the transcriptome response.

This study reports the transcriptomic responses of three salmon species over nine days following exposure to *L. salmonis*. It is likely that the response characterized here would change upon louse development to the later, more aggressively feeding stages, as shown earlier in Atlantic salmon [24]. Additionally, in the present study some genes changed over time independent of exposure status (control or infected). These changes could have been from the exposure or mock exposure of the fish to *L. salmonis* (i.e. reduced water volume and use of the sedative), and indicate the importance of using time-matched controls. Some consistencies in responses were identified in anterior kidney and skin (e.g., antiviral suppression in all species and increased expression of *fkbp5* and *complement C7* in chum salmon). However, the systemic response contained unique aspects relative to the local response. For example, specific to the anterior kidney response was the reduction in iron and heme availability, whereas specific to the skin were pro-inflammatory cytokines *interleukin-1 beta* and *TNF-α*, as well as the antioxidant *thioredoxin*. The inclusion of both systemic (anterior kidney, blood) and local tissues (pectoral fin) in the present work allowed for additional understanding of the organismal response to lice infections, such as iron sequestration in comparison to local inflammation by pro-inflammatory cytokines. Furthermore, the inclusion of both susceptible and refractory species allowed for the comparative characterization of general, susceptible, and resistant responses to lice infections (Table 2).

**Table 2:** **Response functions and relation to susceptibility or resistance**

Function	Response type			
	General	Susceptible	Resistant	Systemic or local
	[A +/or C] + P	[A +/or C] no P	P only	
Unfolded protein response	Y			sys
Acute phase response	Y			sys
Prostaglandin production	Y			sys
Stress-induced and apoptosis	Y			sys/loc
Complement and coagulation	Y			sys/loc
Metalloproteinase activity	Y			sys/loc
Antiviral suppression	Y			sys/loc
Antioxidant activity	Y			loc
Antigen presentation suppression		Y		sys
Reduced hematocrit		Y		sys
Elevated cortisol		Y		sys
Iron homeostasis/heme suppression			Y	sys
Innate pattern recognition receptor			Y	sys
Local inflammation/cytokines			Y	loc

Summarized response types of susceptible (Atlantic and chum) and resistant (pink salmon) separated by evidence of a general response (Atlantic and/or chum and pink), a susceptible response (Atlantic and/or chum and not pink) or a resistant response (pink and not Atlantic or chum). Functions are also identified as being present as a systemic response or local response.

## Conclusions

Multiple experimental infections of Atlantic, chum, and pink salmon indicate highest susceptibility in chum salmon (high infection density, reduction in weight gain and hematocrit, and elevated cortisol), followed by Atlantic salmon (high infection density, reduction in hematocrit), and lowest susceptibility in pink salmon. Differences in susceptibility were observed as early as three days post exposure. General systemic response mechanisms were identified, including cellular protection, acute phase response, complement cascades and pattern recognition receptors. Due to susceptibility differences between chum and pink salmon, comparisons within *Oncorhynchus* were important in understanding potential resistance factors, such as systemic iron sequestration, increased expression of pattern recognition receptor *c-type lectin family 4 member M* and *acidic mammalian chitinase*, as well as local induction of pro-inflammatory *interleukin-1 beta* in pink salmon. Furthermore, in both local and systemic responses of Pacific salmon, up-regulation of lice response genes coincided with suppressed antiviral genes, indicating the importance of investigating co-infection dynamics of salmon responding to both lice and viruses.

## Methods

### Animals and exposure

Pink and chum salmon were obtained as swim-up fry (<0.5 g) from the Quinsam River and Nanaimo River hatcheries, respectively, on Vancouver Island, British Columbia. Atlantic salmon (20–35 g) were obtained from a commercial freshwater hatchery on Vancouver Island. Prior to experimentation, fish were reared in 400 L tanks in flowing water that was an equal mixture of aerated freshwater and seawater and fed a diet of commercial salmon pellets at a daily rate of 1.0% biomass. The photoperiod was regulated to mimic seasonal variation, ranging from 16 light: 8 dark in summer to 8: 16 in winter. Seawater used for fish maintenance and experimentation was pumped from Departure Bay and sand-filtered to approximately 30 μm with a mean salinity of 29.5 ± 0.5‰ and mean dissolved oxygen of 9.5 ± 0.5 mg/L. The seawater temperature displayed seasonal variation as indicated below. Ovigerous *Lepeophtheirus salmonis* were collected from adult Atlantic salmon following harvest from a farm near Vancouver Island and transported in ice cold aerated seawater to Nanaimo. Dissected egg strings were incubated in filtered and ultraviolet irradiated seawater at 9.5 ± 1.0C and 29.5 ± 0.5‰ salinity, with supplemental aeration, as described previously [72]. Cultured lice were monitored by daily microscopic examination of triplicate samples and an inoculum containing a known number of copepodids was prepared when the ratio of copepodid to nauplius II stages was greatest.

Three trials were conducted to characterize the infection over the life cycle of *L. salmonis*. In Trials 1–3, the mean seawater temperature was 10.5, 11.5 and 8.5°C, respectively, reflecting ambient conditions in early November (Trials 1 and 2) and from mid-January to late February (Trial 3). All fish were acclimated to full-strength seawater a minimum of 10 days prior to exposure to *L. salmonis* (see Additional file 2: Table S1 for fish weight). In Trial 1, 10 individuals from each species (approx. 45-70 g) were randomly allocated to each of eight seawater tanks. A total of 5014 copepodids (167/fish) were added to each of four tanks using the metomidate hydrochloride (Aquacalm, Syndel Laboratories Ltd.) sedation exposure method described previously [72]. In Trial 2, 15 individuals of each species (approx. 40-70 g) were randomly allocated to 4 tanks. A total of 7,335 copepodids (163/fish) were added to each of two tanks as described above. In Trial 3, 12–15 individuals of each species (approx. 50-80 g) were randomly allocated to each of four tanks. A total of 8,900 copepodids (199/fish) were added to each of two tanks as described above. In each trial, salmon co-habiting in control tanks were treated the same as exposed fish without the addition of copepodids. In Trial 1, all fish were sampled from one exposed and one control tank at three, six, nine and 12 days post-exposure (dpe). For sampling, salmon were sedated with 0.5 mg/L metomidate, immersed in 200 mg/L MS-222 until immobile and killed with a blow to the head, as previously described [22]. In subsequent trials, fish were sampled as above, but at seven and 14 dpe (Trial 2) and at 28 and 43 dpe (Trial 3). All processing was performed rapidly: each fish was measured for fork length, weight, and lice count and lice were stored in 95% ethanol for later assessment of development stage [72]. Blood was collected from the caudal peduncle into heparinated tubes. In Trial 1, the left pectoral fin and approximately 7 mm of the anterior kidney were rapidly dissected from each fish, flash frozen separately in liquid nitrogen, then stored at −80°C until RNA extraction. In Trial 1, blood was centrifuged (3,000 RPM, 20 minutes) and plasma collected and stored at −80°C for cortisol quantification. For Trial 2 and 3, blood was centrifuged for 3 minutes (11,700 RPM, Autocrit Ultra 3, Becton Dickinson) and hematocrit measured immediately. Use of research animals complied with Fisheries and Oceans Canada Pacific Region Animal Care Committee protocol numbers 06–004 and 09–001.

Total RNA was extracted from fin and kidney samples in Trial 1 using TRIzol (Invitrogen), as per manufacturer’s instructions, and purified using RNeasy spin columns (QIAGEN), by manufacturer’s instructions with the on-column DNase I digestion. The RNA was quality checked by agarose gel electrophoresis and quantified by spectrometry (NanoDrop-1000).

### Cortisol, weight and hematocrit analyses

Cortisol levels in plasma were tested by immunoassay of 20 μl samples (Parameter™, R&D Systems). Samples were run in duplicate, and a standard curve and interplate calibrator sample was run on each plate. All samples were within the high range of the standard curve and the reported minimum detectable limit of the kit (R&D Systems).

For each species, data analysis of cortisol concentration, fish weight, and hematocrit levels were performed using a linear models in the statistical environment R (v2.14.1; [73]) using day and exposure (with interactions) as explanatory variables. Significance between groups was tested using post-hoc Tukey’s HSD tests between conditions of interest.

### cDNA synthesis and microarray preparation

Total RNA samples were randomized and 200 ng total RNA of each sample was reverse transcribed to cDNA and amplified to labelled-cRNA using Low Input Quick Amp labelling kits as per manufacturer’s instructions (Agilent). A reference pool was synthesized using equimolar amounts of Cy3-cRNA from each species/day/infection class condition to hybridize alongside experimental samples to control for hybridization difference in a common reference design [74] (19 samples used in reference pool). Experimental samples (labelled with Cy5) included 9–11 biological replicates for the infected individuals and 9–10 biological replicates for time-matched controls. Anterior kidney samples for Atlantic and pink salmon were compared at days 3, 6 and 9 post infection, and for chum salmon at day 6 only (total number of infected or control samples at all days = 57, 20, and 60 for Atlantic, chum, and pink, respectively). Skin samples were profiled only at day 6 post infection (total number of samples for both infected and time-matched controls = 18, 20 and 19 for Atlantic, chum, and pink, respectively). Samples were hybridized to randomized-order cGRASP 4x44k salmonid arrays using previously reported probe annotation ( [35, 75] Agilent eArray AMADID: 025055) as per manufacturer’s instructions and slides were washed using stabilization solution to minimize ozone-related problems (Agilent; [76]). Slides were kept in the dark in a low ozone atmosphere and scanned on a ScanArray Express (Perkin Elmer) at constant PMT settings to produce saturated median values for ~1% of spots (Cy3:80; Cy5:75). Images were quantified using Imagene (v8; BioDiscovery) and poor or empty spots were flagged. For each spot, the median of the background signal was subtracted from the foreground median. Sample files were then imported into GeneSpring (v11; Agilent), negative raw values were set at 1.0, each array was normalized by intensity-dependent *Lowess* (Agilent; [77]), and a baseline to median transformation of normalized expression values was performed per gene (Agilent). All species and tissues were separately normalized and comparisons between species were indirect.

For each species and tissue experiment, (e.g., Atlantic salmon, anterior kidney) filters were applied to retain probes for which 65% or more of all samples within at least one condition had background-corrected raw expression values ≥ 500 in both channels and flag values for each channel as ‘present’. For statistics tests, a probe was deemed differentially expressed if it passed a Benjamini-Hochberg multiple test corrected p-value ≤ 0.05 and fold change ≥ 1.5. In experiments with a time component (e.g., Atlantic or pink salmon anterior kidney), a 2-way ANOVA was used to detect probes with a significant effect of infection and those with a time-infection interaction effect. Probes with a main effect but no interaction effect were filtered to retain only those that varied by 1.5 fold between control and experimental for at least one of the three time points. Probes with a significant time by infection interaction were filtered at each time point (FC ≥ 1.5). Principal component analysis of samples based on gene expression levels was performed in GeneSpring using a separate analysis from the differential expression analysis. Here all species were normalized together, and probes used only if they passed quality control in all species (Agilent). Enrichment analysis of up- or down-regulated gene lists was performed using Entrez-ID identifiers imported into the DAVID bioinformatics platform [78] using a background list specific to each species (all entities passing quality control filter for each experiment). Overlap between differential lists was evaluated using *VENNY*[79].

### Reverse-transcription quantitative polymerase chain reaction (RT-qPCR)

Purified total RNA used for the microarray experiments was also used to generate cDNA for reverse-transcriptase quantitative polymerase chain reaction (RT-qPCR) using SuperScript III First-Strand Synthesis System for RT-PCR (Invitrogen) as per manufacturer’s instructions. Each cDNA sample was diluted 20-fold. To ensure efficiency in all species and tissues, a standard curve was generated for each species and tissue (n = 6 dilution series) using pooled equimolar amounts from three samples from each condition, diluting the pool 10-fold and then producing a 5-fold dilution series (six points). All primers had efficiency values within the range of 90-110% for all three species. qPCR amplification was performed using SsoFast™ EvaGreen® (Bio-Rad) in 20 μL reactions in an MX3000P (Agilent) as previously described [76], with the exception of running triplicate technical replicates. Genes of interest were selected based on relevance to the study system, presence in enriched functional categories, high significance or fold change, and relevance to multiple tissues. Reference candidates were selected based on other studies, unchanging expression in infected/control individuals in microarray analysis and moderate expression levels in all three species and tissues. Primers were designed in Primer3 [80] selecting amplicon sizes of 80–150 bases. Amplicons were checked for single products by melt curve analysis, and were sequenced to confirm identity as previously described [10].

Data analysis was performed using qbasePLUS (Biogazelle) and reference gene stability was tested using geNorm [81]. The three most stable reference genes chosen for the current analysis in all species and all tissues were *dynein light chain 1 cytoplasmic*, *U6 snRNA-associated Sm-like protein lsm8*, and *mRNA turnover protein 4 homolog* with collective M (and CV) values for Atlantic, chum, and pink anterior kidney and skin of 0.321(0.129) and 0.349(0.141), 0.413(0.175) and 0.421(0.455), and 0.254(0.101) and 0.280(0.111), respectively. These values are within the range typically observed for stably expressed reference genes in heterogeneous samples [82]. A minimum of 2 technical replicates were found to be within 0.5 Ct for all samples. The interplate calibrator used to compare across plates within a gene had a <0.5 Ct difference for all genes within each species. NTC and -RT controls showed no amplification. Significance for Atlantic and pink salmon anterior kidney was determined by two-way ANOVA, and for all other infected/control comparisons with only one time point by *t-*test in R [73]. Statistics were performed on log_10_ transformed data. Correlation between methods were checked using linear best fit lines of log_2_ expression values for samples measured by RT-qPCR against microarray (using the microarray probe corresponding to the contig used for primer design).

Several immune system genes not present on the array, but identified as louse response genes [33, 83] were included in an additional qPCR analysis including *interleukin-1 beta*, *interleukin-8*, *prostaglandin D synthase* and *tumour necrosis factor alpha*. For these immune genes, a randomly selected subset of the samples used for the full study were used to test for expression differences (n = 5–7 samples per condition). As *dynein light chain 1 cytoplasmic* (*dynll1*) was already found to be stable for these samples (above), expression was normalized using the geometric mean of *dynll1* and *eukaryotic translation initiation factor 4H*. Each primer pair was evaluated for each species individually as described above except that only one tissue was tested for efficiency, and standard curves were approximately in the range of the sample values. All technical replicates were within 0.5 Ct for 224/228 combinations. Primer thermal regimes for these genes were reported previously [33].

### Data accessibility

Gene expression data files have been uploaded to Gene Expression Omnibus (GSE48337).

## Electronic supplementary material

Additional file 1: Figure S1.: Louse development rates on all species. Development stages of lice as a percentage of the total lice found per day on each species for Trial 1 **(A)**, and Trials 2 and 3 **(B)**. (PDF 334 KB)

Additional file 2: Table S1: Fish weight and infection prevalence/intensity. (XLSX 13 KB)

Additional file 3: Figure S2: Multiple species utility of microarray. (A) When all species are normalized together, principal components analysis (PCA) indicates the largest variance between genus *Salmo* (PC1+) and *Oncorhynchus* (PC1-), and the second largest variance between species *O. keta* (PC2-) and *O. gorbuscha* (PC2+). The basal expression differences captured by the PCA are due to both true biological differences and technical differences in probe hybridization efficiency between species. (B) When each species is normalized individually (6 dpe only) a similar quantity and identity passed quality control thresholds in all three species, with 5553 uniquely annotated transcripts present in all three species (union set of the Venn diagram). During differential expression testing, each species was therefore normalized separately, and indirectly compared. *Data shown*: *anterior kidney.* (PDF 350 KB)

Additional file 4: Table S2: Primers for qPCR. (XLSX 12 KB)

Additional file 5: Table S3: Differentially expressed gene lists. (XLSX 270 KB)

Additional file 6: Figure S3: Differentially expressed cellular stress, prostaglandin, coagulation and other related genes. Differentially expressed genes involved in response to cellular stress, prostaglandin metabolism, FK506-binding, coagulation and other related functions displayed with linear fold change values for each day (D3-D9) and colored by fold change (FC) relative to controls (green = down-regulated; red = up-regulated). Bold values indicate highly significant main effect of infection (p < 0.0001), asterisks indicate significant time by infection interaction, and italics indicates no significant main effect (significant interaction only). A hyphen indicates no significant difference identified and an ‘x’ indicates no probe passing quality control for the species. (PDF 713 KB)

Additional file 7: Figure S4: Differentially expressed immunity genes. Differentially expressed genes involved in antiviral response, and other immune-related functions. Colors and formats are as described in Additional file 6: Figure S3. (PDF 690 KB)

Additional file 8: Figure S5: Differentially expressed genes in chum salmon skin. Selected differentially expressed genes in the skin of chum salmon at 6 days post exposure involved in immunity, proliferation, and other functions. Antiviral genes are suppressed as is seen in the anterior kidney of both Pacific salmon. Colors and formats are as described in Additional file 6: Figure S3. (PDF 438 KB)

Additional file 9: Figure S6: qPCR microarray log_2_ expression correlation. **(A)** Microarray and qPCR expression levels correlated well for all significantly differentially expressed genes in the anterior kidney for all three species. Skin sample correlation was lower, but still always identified the correct direction of fold change. Primers were designed to ensure equal amplification for all species to ensure correct estimates of expression levels, as shown for *collagenase-3* log2(qPCR) against log2(microarray) shown for **(B)** Atlantic and **(C)** pink salmon. chm = chum; pnk = pink; atl = Atlantic; AK = anterior kidney; S = skin; gene acronyms are as per the primer table (Additional file 4: Table S2). (PDF 390 KB)

Additional file 10: Figure S7: Expression of *collagenase-3* and *15-hydroxyprostaglandin dehydrogenase* by qPCR. (A) *Collagenase-3* expression in the anterior kidney evaluated by qPCR. (B) Expression of the prostaglandin E_2_ inactivator *15-hydroxyprostaglandin dehydrogenase* was suppressed relative to the control in the anterior kidney of all three species early in the infection. Boxplot displays median and interquartile range, and circles are outliers. *denotes p < 0.05; **denotes p < 0.001. (PDF 366 KB)
