# Supplementary figures and images for: Comparative transcriptomics of Atlantic Salmo salar, chum Oncorhynchus keta and pink salmon O. gorbuscha during infections with salmon lice Lepeophtheirus salmonis
Source: BMC Genomics. 2014 Mar 15;15(1):200. doi: 10.1186/1471-2164-15-200 (PMC4004277; doi:10.1186/1471-2164-15-200)

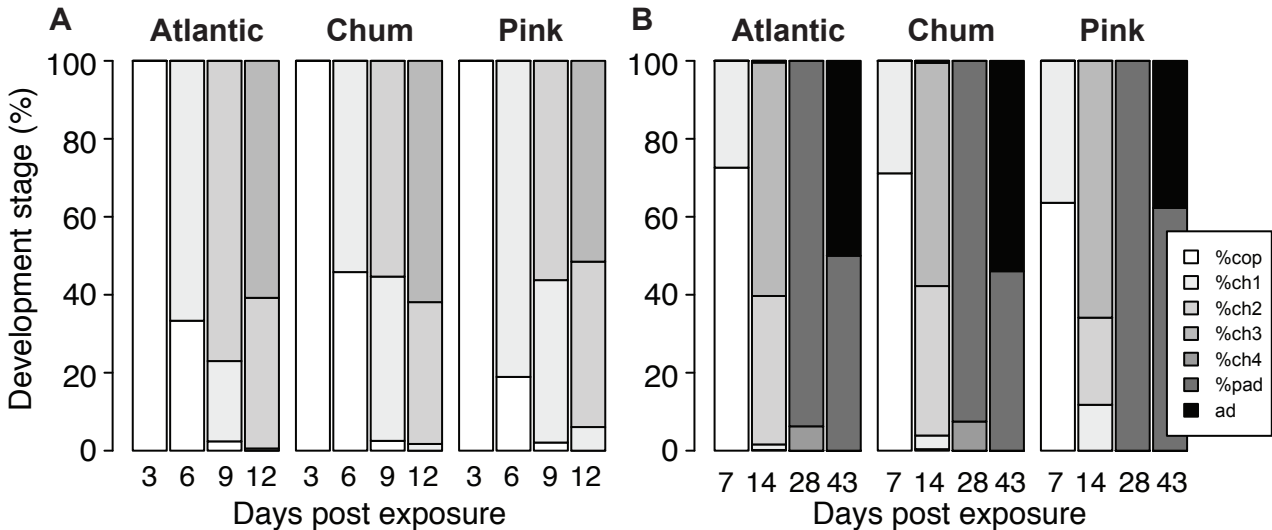

Supplement: Supplementary file 1 — Additional file 1: Figure S1.: Louse development rates on all species. Development stages of lice as a percentage of the total lice found per day on each species for Trial 1 (A), and Trials 2 and 3 (B). (PDF 334 KB) [file 12864_2013_7038_MOESM1_ESM.pdf]

**A**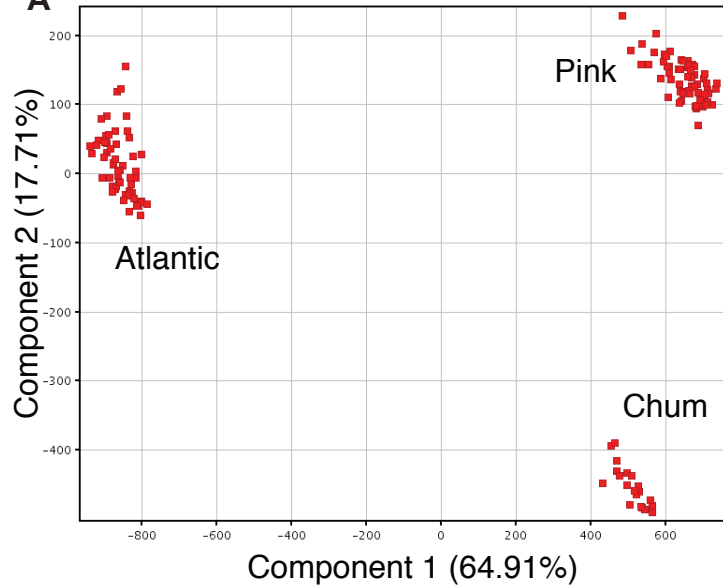**B**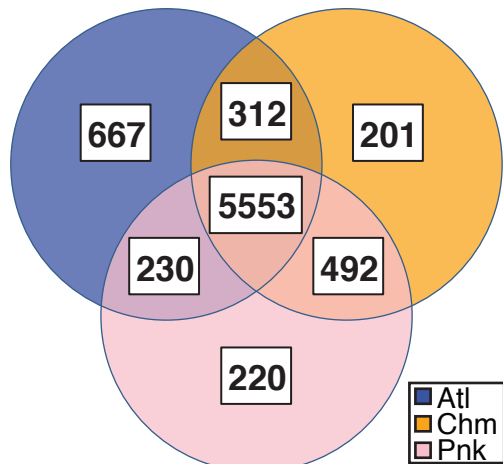

Supplement: Supplementary file 3 — Additional file 3: Figure S2: Multiple species utility of microarray. (A) When all species are normalized together, principal components analysis (PCA) indicates the largest variance between genus Salmo (PC1+) and Oncorhynchus (PC1-), and the second largest variance between species O. keta (PC2-) and O. gorbuscha (PC2+). The basal expression differences captured by the PCA are due to both true biological differences and technical differences in probe hybridization efficiency between species. (B) When each species is normalized individually (6 dpe only) a similar quantity and identity passed quality control thresholds in all three species, with 5553 uniquely annotated transcripts present in all three species (union set of the Venn diagram). During differential expression testing, each species was therefore normalized separately, and indirectly compared. Data shown: anterior kidney. (PDF 350 KB) [file 12864_2013_7038_MOESM3_ESM.pdf]

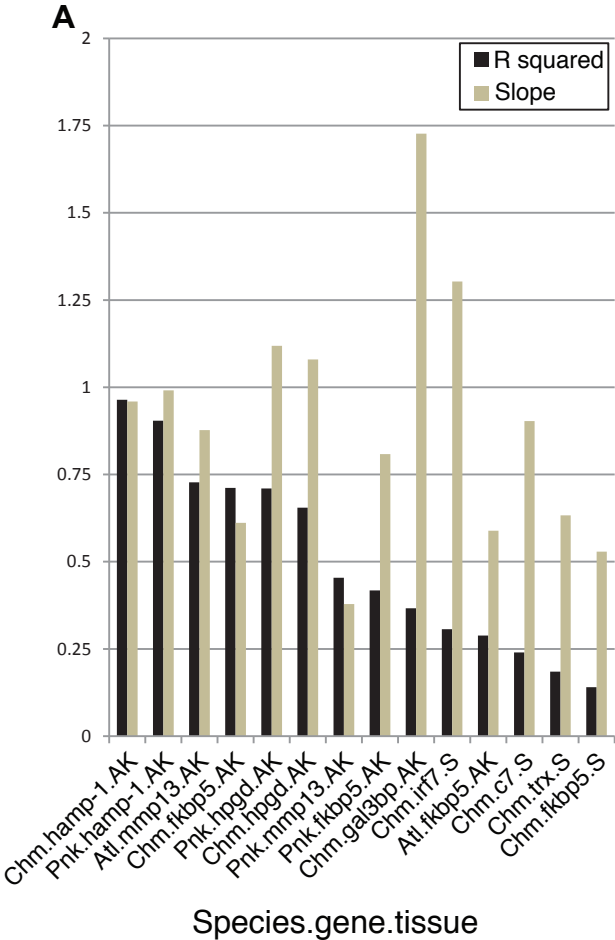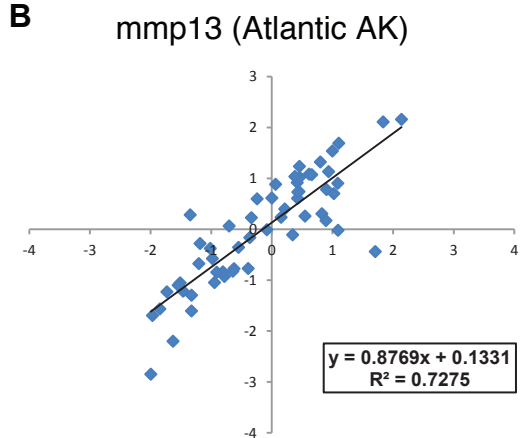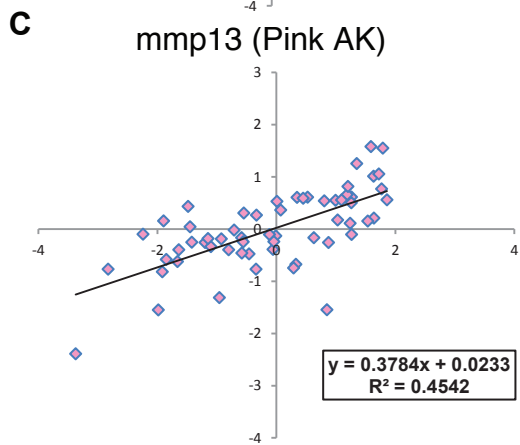

Supplement: Supplementary file 9 — Additional file 9: Figure S6: qPCR microarray log2 expression correlation. (A) Microarray and qPCR expression levels correlated well for all significantly differentially expressed genes in the anterior kidney for all three species. Skin sample correlation was lower, but still always identified the correct direction of fold change. Primers were designed to ensure equal amplification for all species to ensure correct estimates of expression levels, as shown for collagenase-3 log2(qPCR) against log2(microarray) shown for (B) Atlantic and (C) pink salmon. chm = chum; pnk = pink; atl = Atlantic; AK = anterior kidney; S = skin; gene acronyms are as per the primer table (Additional file 4: Table S2). (PDF 390 KB) [file 12864_2013_7038_MOESM9_ESM.pdf]

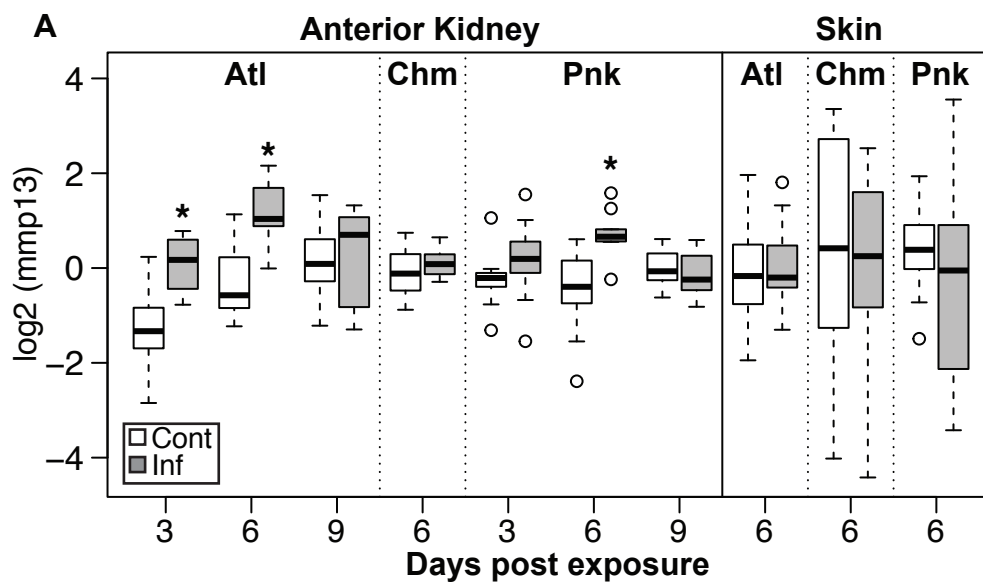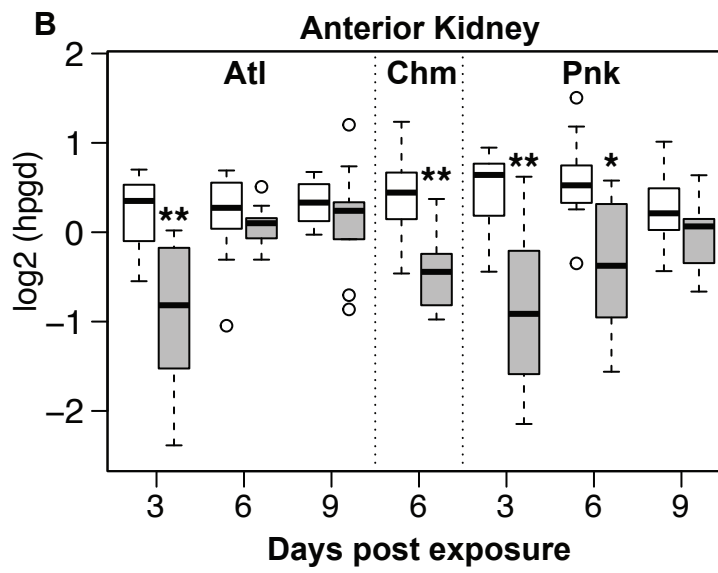

Supplement: Supplementary file 10 — Additional file 10: Figure S7: Expression of collagenase-3 and 15-hydroxyprostaglandin dehydrogenase by qPCR. (A) Collagenase-3 expression in the anterior kidney evaluated by qPCR. (B) Expression of the prostaglandin E2 inactivator 15-hydroxyprostaglandin dehydrogenase was suppressed relative to the control in the anterior kidney of all three species early in the infection. Boxplot displays median and interquartile range, and circles are outliers. *denotes p < 0.05; **denotes p < 0.001. (PDF 366 KB) [file 12864_2013_7038_MOESM10_ESM.pdf]
