# Supplementary material for: Comparative transcriptomics of Atlantic Salmo salar, chum Oncorhynchus keta and pink salmon O. gorbuscha during infections with salmon lice Lepeophtheirus salmonis
Source: BMC Genomics. 2014 Mar 15;15(1):200. doi: 10.1186/1471-2164-15-200 (PMC4004277; doi:10.1186/1471-2164-15-200)
Supplement: Supplementary file 6 — Additional file 6: Figure S3: Differentially expressed cellular stress, prostaglandin, coagulation and other related genes. Differentially expressed genes involved in response to cellular stress, prostaglandin metabolism, FK506-binding, coagulation and other related functions displayed with linear fold change values for each day (D3-D9) and colored by fold change (FC) relative to controls (green = down-regulated; red = up-regulated). Bold values indicate highly significant main effect of infection (p < 0.0001), asterisks indicate significant time by infection interaction, and italics indicates no significant main effect (significant interaction only). A hyphen indicates no significant difference identified and an ‘x’ indicates no probe passing quality control for the species. (PDF 713 KB) [file 12864_2013_7038_MOESM6_ESM.pdf]

FUNCTION	GENE	Atlantic D3	Atlantic D6	Atlantic D9	Chum D6	Pink D3	Pink D6	Pink D9
Cellular stress and apoptosis	Programmed cell death protein 10	-	-	-	-	1.65	-1.02	1.21
	Stress-induced-phosphoprotein 1	2.11	-1.04	1.54	-	2.30	1.53	1.25
	Growth arrest and DNA-damage-inducible protein GADD45 alpha	-	-	-	-	1.46	1.51	1.13
	DNA-damage-inducible transcript 4-like protein	-	-	-	**9.27**	1.05	2.43	1.40
	Apoptosis-inducing factor 2	-	-	-	-	-1.08*	1.62*	1.08*
	TP53-regulated inhibitor of apoptosis 1	-	-	-	-	_-1.22_*	_1.69_*	_1.02_*
	Stress-associated endoplasmic reticulum protein 1	-	-	-	2.09	-	-	-
	BCL2/adenovirus E1B 19 kDa protein-interacting protein 3	-1.05*	3.13*	1.55*	-	-	-	-
	Stress-70 protein, mitochondrial precursor	1.54	1.15	1.10	-	-	-	-
	Growth arrest-specific protein 8	-1.24	-1.22	-1.62	-	-	-	-
	p53 apoptosis effector related to PMP-22	-1.27	-1.30	-1.75	-	-	-	-
	Apoptosis inhibitor 5	1.58	1.08	1.10	-	-	-	-
	Cyclin-dependent kinase 4 inhibitor B	1.00*	4.15*	1.90*	-	x	x	x
	Cyclin-dependent kinase inhibitor 1C	_-1.44_*	_1.72_*	_1.16_*	-	x	x	x
	Cyclin-dependent kinase inhibitor 1	x	x	x	1.61	_-1.55_*	_1.52_*	_1.20_*
	G1/S-specific cyclin-D1	-	-	-	-	1.00	-1.70	-1.58
Prostaglandins and leukotrienes	Prostaglandin E synthase 3	2.12	1.18	1.16	1.83	**1.34**	**1.75**	**1.21**
	15-hydroxyprostaglandin dehydrogenase [NAD+]	-	-	-	-1.56	**-2.29***	**-1.54***	**-1.06***
	NADP-dependent leukotriene B4 12-hydroxydehydrogenase	-	-	-	-	-1.17	2.17	1.48
	Leukotriene B4 receptor 1	1.83	1.41	-1.02	-1.61	x	x	x
FK506-binding	FK506-binding protein 2	-	-	-	**1.66**	-	-	-
	FK506-binding protein 5	2.28	1.78	1.05	**4.81**	2.41*	1.66*	-1.44*
Coagulation	Plasminogen activator inhibitor 1	1.71	1.36	1.48	-	1.86	2.1	1.35
	Tissue-type plasminogen activator	1.51*	3.33*	1.17*	-	-	-	-
	Tissue factor pathway inhibitor 2	-	-	-	-	1.55	1.37	1.05
	Plasminogen	1.7	1.46	2.29	-	-	-	-
	Coagulation factor X	-	-	-	-1.73	-	-	-
	Platelet-activating factor acetylhydrolase	-	-	-	**-2.06**	-1.36	-1.59	-1.24
	Alpha-2-macroglobulin	-	-	-	-	-1.14	-1.66	-1.05
	CD9 antigen	-	-	-	-1.65	-2.18	-1.37	-1.21
Iron-related, hemopoesis, and other related functions	Putative ferric-chelate reductase 1	-1.33	-2.78	-2.64	-	-	-	-
	Ferritin, middle subunit	_-1.73_*	_3.98_*	_-1.25_*	-	-	-	-
	Iron/zinc purple acid phosphatase-like protein	-1.86	-1.13	-1.71	-	-	-	-
	Iron-sulfur cluster assembly 2 homolog, mitochondrial	_-1.51_*	_1.28_*	_-1.30_*	x	-	-	-
	T-cell acute lymphocytic leukemia protein 1 homolog	_-1.14_*	_2.26_*	_1.14_*	-	-1.17	-1.45	-1.57
	Interleukin-20 receptor alpha chain	-	-	-	-1.98	**-1.74**	**-1.71**	**-2.24**
	Peroxisomal proliferator-activated receptor A-interacting complex 285 kDa protein	-	-	-	**-3.79**	**-1.89**	**-2.02**	**-1.26**
	S-antigen protein	-	-	-	x	1.05	2.62	1.74
	Interferon-stimulated 20 kDa exonuclease-like 1	_1.09_*	_1.56_*	_-1.08_*	-	-	-	-
	TRAF and TNF receptor-associated protein homolog	-	-	-	-1.83	**-1.70**	**-1.67**	**-1.31**
	Nuclear factor interleukin-3-regulated protein	-	-	-	-	2.10	1.04	1.11
