# Supplementary material for: Comparative transcriptomics of Atlantic Salmo salar, chum Oncorhynchus keta and pink salmon O. gorbuscha during infections with salmon lice Lepeophtheirus salmonis
Source: BMC Genomics. 2014 Mar 15;15(1):200. doi: 10.1186/1471-2164-15-200 (PMC4004277; doi:10.1186/1471-2164-15-200)
Supplement: Supplementary file 7 — Additional file 7: Figure S4: Differentially expressed immunity genes. Differentially expressed genes involved in antiviral response, and other immune-related functions. Colors and formats are as described in Additional file 6: Figure S3. (PDF 690 KB) [file 12864_2013_7038_MOESM7_ESM.pdf]

FUNCTION	GENE	Atlantic D3	Atlantic D6	Atlantic D9	Chum D6	Pink D3	Pink D6	Pink D9
Antiviral	Galectin-3-binding protein	-	-	-	-1.86	**-1.8**	**-1.58**	**-1.55**
	Interleukin-1 receptor-associated kinase 3	-1.25	-1.67	-1.16	-	-	-	-
	Interferon regulatory factor 1	-	-	-	-	**-1.54**	**-1.65**	**-1.21**
	Interferon regulatory factor 3	-	-	-	-1.77	-1.36	-1.57	-1.28
	Interferon regulatory factor 7	-	-	-	-1.81	**-2.50**	**-2.05**	**-1.75**
	Interferon-induced GTP-binding protein Mx	-	-	-	-3.65	**-1.95**	**-2.09**	**-1.48**
	Interferon-induced guanylate-binding protein 1	-	-	-	-	**-1.57**	**-1.48**	**-1.61**
	Interferon-induced guanylate-binding protein 2	x	x	x	-	-1.30	-1.50	-1.08
	Interferon-induced 35 kDa protein homolog	-	-	-	-	**-1.62**	**-1.30**	**-1.35**
	Interferon-induced protein 44	1.95	1.22	3.82	**-4.47**	**-2.15**	**-2.54**	**-1.63**
	Interferon-induced protein with tetratricopeptide repeats 5	-	-	-	-1.89	-	-	-
	Interferon-induced very large GTPase 1	-	-	-	-2.41	-1.88	-1.65	-1.26
	Interferon-induced, double-stranded RNA-activated protein kinase	-	-	-	-2.01	-1.69	-1.45	-1.33
	Interleukin-1 receptor type II	-	-	-	x	1.54	1.35	-1.14
	Tripartite motif-containing protein 16	-	-	-	-2.30	-1.45	-1.63	-1.18
	Tripartite motif-containing protein 25	-	-	-	-2.16	-1.40	-1.55	-1.21
	Tripartite motif-containing protein 29	-	-	-	-1.71	-	-	-
	Tripartite motif-containing protein 39	-	-	-	-	**-1.84**	**-1.48**	**-1.43**
	Sacsin	-	-	-	**-3.45**	-1.64	-1.84	-1.26
	Signal transducer and activator of transcription 1	-	-	-	-2.13	**-2.20**	**-2.74**	**-1.74**
	Signal transducer and activator of transcription 1-alpha/beta	-	-	-	-1.67	**-2.17**	**-1.44**	**-1.51**
	Barrier-to-autointegration factor	-1.51*	1.17*	1.14*	-2.16	-	-	-
Antigen presentation	H-2 class II histocompatibility antigen gamma chain	-	-	-	-1.84	-	-	-
	H-2 class II histocompatibility antigen, A-K beta chain	-3.36	1.11	-1.49	-1.64	-	-	-
	H-2 class II histocompatibility antigen, E-S beta chain	-2.30*	1.07*	-1.07*	**-1.58**	-	-	-
	H-2 class II histocompatibility antigen, I-A beta chain	-2.52*	1.23*	-1.18*	-	-	-	-
	HLA class II histocompatibility antigen, DP alpha chain	-	-	-	-1.58	-	-	-
	RT1 class II histocompatibility antigen, B alpha chain	-1.72	-1.26	-1.31	x	x	x	x
	Gamma-interferon-inducible lysosomal thiol reductase	-1.65	-1.11	-1.75	-	-	-	-
Cell-mediated immunity	Beta-2-microglobulin	-	-	-	**-1.72**	-	-	-
	Ig mu chain C region membrane-bound form	-	-	-	2.27	-	-	-
	Interleukin enhancer-binding factor 2 homolog	-	-	-	-	1.13*	1.50*	1.05*
	T-cell immunoglobulin and mucin domain-containing protein 4	-	-	-	-1.72	-	-	-
	T-cell surface glycoprotein CD3 zeta chain	-	-	-	-	-1.20	-1.85	1.01
	CD83 antigen	-	-	-	-1.72	-	-	-
	CD97 antigen	-	-	-	-1.84	-	-	-
	CD276 antigen	-	-	-	1.72	-	-	-
Chemoattraction	Leukocyte cell-derived chemotaxin 2	-	-	-	-	**-2.26***	**-1.01***	**-1.3***
	High affinity interleukin-8 receptor B	1.72	2.38	1.22	-	-	-	-
	Galectin-9	-	-	-	-1.91	-1.52	-1.26	-1.16
