# Supplementary material for: Comparative transcriptomics of Atlantic Salmo salar, chum Oncorhynchus keta and pink salmon O. gorbuscha during infections with salmon lice Lepeophtheirus salmonis
Source: BMC Genomics. 2014 Mar 15;15(1):200. doi: 10.1186/1471-2164-15-200 (PMC4004277; doi:10.1186/1471-2164-15-200)
Supplement: Supplementary file 8 — Additional file 8: Figure S5: Differentially expressed genes in chum salmon skin. Selected differentially expressed genes in the skin of chum salmon at 6 days post exposure involved in immunity, proliferation, and other functions. Antiviral genes are suppressed as is seen in the anterior kidney of both Pacific salmon. Colors and formats are as described in Additional file 6: Figure S3. (PDF 438 KB) [file 12864_2013_7038_MOESM8_ESM.pdf]

FUNCTION	GENE	FC
Immunity	Complement component C7	1.89
Proliferation and differentiation	Growth hormone receptor	1.75
	Adseverin	2.63
	Fibroblast growth factor-binding protein 1	2.58
Other Functions	Breast cancer anti-estrogen resistance protein 1	2.10
	FK506-binding protein 5	4.31
	Growth arrest and DNA-damage-inducible protein GADD45 beta	2.88
	P2X purinoceptor 1	1.95
	Thioredoxin	2.73
Antiviral	Barrier-to-autointegration factor	-1.98
	Beta-2-microglobulin	-2.34
	Galectin-3-binding protein	-2.03
	H-2 class II histocompatibility antigen, A-K beta chain	-1.75
	H-2 class II histocompatibility antigen, E-S beta chain	-2.16
	HLA class II histocompatibility antigen, DP alpha chain	-2.28
	Interferon regulatory factor 3	-1.89
	Interferon regulatory factor 7	-2.09
	Interferon-induced 35 kDa protein homolog	-1.65
	Interferon-induced GTP-binding protein Mx	-2.61
	Interferon-induced guanylate-binding protein 1	-2.53
	Interferon-induced guanylate-binding protein 1	-2.27
	Interferon-induced protein 44	-4.02
	Interferon-induced very large GTPase 1	-2.55
	Lymphocyte cytosolic protein 2	-1.70
	Radical S-adenosyl methionine domain-containing protein 2	-4.42
	Sacsin	-3.66
	Signal transducer and activator of transcription 1	-2.55
	T-cell receptor alpha chain V region HPB-MLT	-1.80
	Tripartite motif-containing protein 16	-1.84
	Tripartite motif-containing protein 25	-1.95
Other Functions	Interleukin-20 receptor alpha chain	-4.33
	Peroxisomal proliferator-activated receptor A-interacting complex 285 kDa protein	-3.33
	Probable E3 ubiquitin-protein ligase HERC4	-2.33
	Thrombospondin-2	-1.84
